# Health impacts and risk assessment of PM_2.5_ and PM_10_ at Suburban Site in Pathum Thani, Thailand

**DOI:** 10.1016/j.toxrep.2025.102109

**Published:** 2025-08-08

**Authors:** Dussadee Rattanaphra, Sittinun Tawkaew, Wilasinee Kingkam, Sasikarn Nuchdang, Kittiwan Kitpakornsanti, Unchalee Suwanmanee

**Affiliations:** a Nuclear Technology Research and Development Center, Thailand Institute of Nuclear Technology, Nakorn Nayok 26120, Thailand; b Department of Chemical Engineering, Faculty of Engineering, Srinakharinwirot University, Nakorn Nayok 26120, Thailand; c Climate Change and Environment Research Center, Department of Climate Change and Environment, Technopolis, Pathumthani 12120, Thailand

**Keywords:** PM₂.₅, PM₁₀, Enrichment factor, Risk assessment, Carcinogenic, Non-carcinogenic, Pathum Thani Thailand

## Abstract

The aim of this study was to evaluate the carcinogenic and non-carcinogenic health risks associated with heavy metals in PM₂.₅ and PM₁₀ through inhalation exposure among children and adults during both the summer and wet seasons in the Pathum Thani Province, Thailand. PM2.5 and PM10 samples were collected using a Tisch TE-Wilbur sampler, and elemental concentrations were analyzed using Proton-Induced X-Ray Emission (PIXE). Microsoft Excel was employed to determine the statistical values of PM₂.₅ and PM₁₀ concentrations, the concentrations of twelve elements, including Si, S, Cl, K, Ca, Ti, Cr, Mn, Fe, Zn, Ni, and Cu. The enrichment factor (EF), as well as health risk assessment indicators, including target hazard quotient (THQ), hazard index (HI), and carcinogenic risk (CR), were evaluated. The results showed that EF values for Zn, Ni, and Cu ranged from 10 to 100, indicating contributions from anthropogenic sources. Cr exhibited the highest EF values, ranging from 51 to 111, suggesting significant influence from industrial activities and traffic emissions. The mean PM₁₀ concentration (86.0504 µg/m³) during the wet season exceeded the WHO and EU standards but remained below the Thailand standard and the U.S. EPA limit. In contrast, the mean PM₂.₅ concentration (77.5143 µg/m³) during the same period exceeded all referenced standards. The calculated HI values were from 0.0459 to 0.1090 for adults and 0.3285–0.7811 for children. The CR values in PM₂.₅ ranged from 5.0884 × 10⁻⁸ to 7.9544 × 10⁻⁶ for adults and from 5.9364 × 10⁻⁸ to 9.2802 × 10⁻⁶ for children. For PM₁₀, the CR values ranged from 5.1865 × 10⁻⁸ to 1.0412 × 10⁻⁵ for adults and from 6.0509 × 10⁻⁸ to 1.2148 × 10⁻⁵ for children. Although both carcinogenic and non-carcinogenic risks were within acceptable limits, higher risk values were observed in children compared to adults. Therefore, targeted and effective air pollution control policies are recommended, with a particular emphasis on protecting children’s health and strengthening evidence-based air quality management strategies.

## Highlights

• PM₂.₅ concentration (77.51 μg∙m^−3^) during wet season exceeded the Thailand standard (50 μg∙m^−3^).
• The enrichment factors (EFs) of Zn, Ni, Cu, and Cr indicated anthropogenic sources.
• Hazard index values were less than 1, indicating negligible non-carcinogenic health risk.
• Total carcinogenic risks (TCR) were less than 10^−6^, indicating negligible carcinogenic health risk.
• The higher risk values from metal exposure in PM were observed in children compared to adults.

## Graphical Abstract

**Figure:**
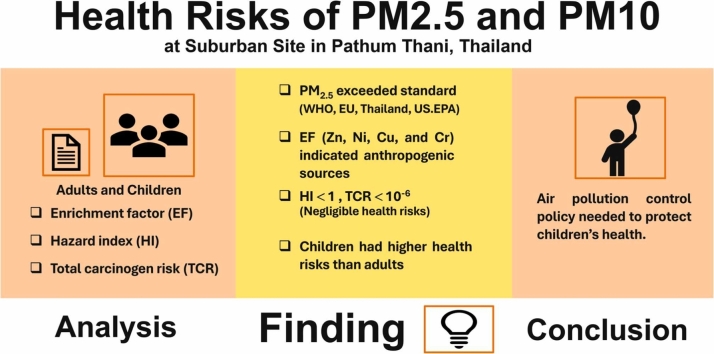

## 1 Introduction

Particulate matter (PM) is an important indicator of air pollution that can be harmful to human health [1]. It generally consists of microscopic solid particles or liquid droplets that can be inhaled and cause serious health problems [2]. Particulate pollution is typically classified into two types: PM₁₀, which includes inhalable particles with diameters of 10 micrometers or less, and PM₂.₅, which consists of fine inhalable particles with diameters of 2.5 micrometers or less [2]. PM₂.₅ and PM₁₀ are released into the environment through various natural pathways (e.g., biomass burning, soil dust resuspension) and anthropogenic pathways (e.g., vehicular emissions, industrial processes, agricultural activities) [4]. The World Health Organization (WHO) reported that 99 % of the global population exposed air that exceeded the WHO’s guideline limits. The combined effects of ambient and household air pollution were estimated to have caused 6.7 million premature deaths globally in 2022 [3]. Moreover, UNICEF [5] reported that a staggering 500,000 of child deaths were linked to household air pollution because of cooking indoors with polluting fuels, primarily in Africa and Asia. This is because of exposure to particulate matter, which causes cardiovascular and respiratory disease, and cancers.

To concern health risk, protect populations, and highlight the need for countries to implement standards and policies to mitigate air pollution, the WHO updated the global air quality guideline in 2021 with the lower annual PM₂.₅ and PM10 levels of 5 μg∙m^−3^ and 10 μg∙m^−3^ [5], compared to the previous guidelines of 15 μg∙m^−3^ and 20 μg∙m^−3^ published in 2005 [6], respectively.

From 2014–2023, air pollution levels in Thailand were exceeded both national (15 μg∙m^−3^ for PM_2.5_ and 50 μg∙m^−3^ for PM₁₀) and WHO (5 μg∙m^−3^) standards. In 2023, the value was moderately increased by 30 % compared to 2022, indicating awareness in PM pollution [7]. Numerous previous studies have investigated the health effects associated with exposure to fine PM in Thailand [8], [9], [10], [11], [12]. Air pollution remains a significant issue in the country, with recent research reporting elevated PM levels in several provinces. In the central region, including Bangkok and Pathum Thani, studies have been conducted by Fold et al. [8], Ahmad et al. [9], and Nuchdang et al. [13]. In the northeast, Nakhon Ratchasima was examined by Dutta et al. [14]. In the northern region, multiple provinces including Chiang Mai, Lamphun, Lampang, Phrae, Nan, Phayao, Chiang Rai, and Mae Hong Son have been investigated by Jarernwong and Gheewala [15] and Supasri et al. [11]. Over the past decades, the health impacts of PM exposure have become a major focus of research not only in Thailand but also in other regions, including the United States, Europe, and Asia. There have been many comprehensive studies on PM and toxic elements in PM in Asia, particularly in China [16].

There are many research in Asia countries, including Thailand, Saudi Arabia, China, and Pakistan studied on PM and toxic elements. In Thailand, Ahmad et al. [9] investigated the characteristics of twenty-two toxic metals (Al, As, Ba, Ca, Ce, Co, Cr, Cu, Cd, Fe, K, Mg, Mn, Na, Ni, Pb, Pt, Sc, Se, Ti, V, and Z) in PM_2.5_ and PM_10_ and health risk in the three inner city (Ari, Din Dang, and Bangna) of Bangkok. The daily concentrations of PM_2.5_ were lower than the permission limit but PM_10_ exceeded the WHO standard. The excess acceptable limits of carcinogen risk were found in adults at Din Dang, and Bangna. The principal component analysis (PCA) indicated that PM_2.5_ and PM_10_ were from anthropogenic sources such as vehicle and industry emissions. In Saudi Arabia, Al-Swadi et al. [17] assessed the characteristics of eleven toxic metals (Al, Fe, Mn, Zn, Ti, Cu, Cr, Co, Ni, Pb, and Cd) in dust and soil with level of health risks from mining area. The finding found that the concentrations of dust and non-carcinogenic risk were higher the standard. The enrichment factor (EF) and PCA analysis indicated that of Cd, Ci, Pb, and Zn, toxic metals were from anthropogenic sources. In China, Zhou et al. [18] studied the characteristics of eleven toxic metals (Al, As, Cr, Hg, Pb, Mn, Ni, Sb, Se, and Tl) in PM_2.5_ and health risk in Suzhou from 2019 to 2021. The average PM_2.5_ concentration during three years (46.76 μg∙m^−3^) was exceeded both China (35 μg∙m^−3^) and WHO (5 μg∙m^−3^) standards. The EF analysis indicated that toxic metals was from anthropogenic sources and the total non-carcinogenic risk values exceeded acceptable thresholds. In Pakistan, Rehman et al. [19] investigated the characteristics of eleven toxic metals (As, Cd, Co, Cr, Cu, Hg, Mo, Ni, Pb, Sb, and Zn) in PM and health risks from eight roads in Lahore. The study found that the concentrations of Cd, Hg, and Mo in PM exceeded WHO standards and health risks.

In Europe and America, the several related previous studies were reported as follows. In Italy, Vaio et al. [20] assessed the characteristics of seven toxic metals (Al, As, Cd, Co, Cr, Fe, Mn, Ni, Pb, Cu, Sb, V, and Zn) in PM_10_ and health risk in Acerra. The daily PM_10_ concentration exceeded the Italy standard in all seasons, except for autumn. The excess acceptable limits of total non-carcinogen risks were found both adults and children. The EF and PCA analysis indicated that of Cd, Sb, Pb, As, Cu, and Zn, toxic metals were from anthropogenic sources such as vehicle and industrial combustions. In Mexico, Breton et al. [21] studied the characteristics of seven toxic metals (Cu, Co, Zn, Cd, Fe, Mg, and Mn) in PM_10_ and health risk in Leon. The average PM_2.5_ concentration during warm and cold seasons (56.42 and 95.97 μg∙m^−3^) exceeded the Mexico standard. The excess acceptable limits of carcinogen and non-carcinogen risks were found in adults and children. The factor analysis indicated that of Cd, Cu, Mg, and Zn, toxic metals were from anthropogenic sources. In Canada, Fakhri et al. [22] investigated the characteristics of nineteen toxic metals (Al, Cu, Co, Cr, Cd, Fe, K, Mg, Mn, Mo, Na, Ni, Pb, Sb, Pb, Sr, Ti, V, and Zn) in PM_2.5_ and health risk in Montreal, Canada. The average daily PM_2.5_ concentration (4 ± 3 μg∙m-3) was lower than the both Canada (27 μg∙m^−3^) and WHO (15 μg∙m^−3^) standards. The carcinogenic risk from Co and Cr(VI) were higher than 10^−6^, indicating this area need more attention to these trace metals.

Yang et al. [23] and Strak et al. [24] reported that although the concentration of PM has shown a downward trend in China, due to fulfilling National Determined Contributions and continuing air pollution control policies, but it did not significantly mitigate the associated health risk. Not only studies on hazardous heavy metal levels and potential health risks from PM, but also the analysis of PM concentrations, provide valuable information to support the development of effective policies and informs policymakers in the specific study area. Pathum Thani Province is located in the central region of Thailand, near Bangkok. The Gross Provincial Product (GPP) per capita is 230,401 THB, ranking 13th in the country and 3rd in the Bangkok Metropolitan Region after Bangkok and Samut Prakan. In 2018–2021, the Pollution Control De-partment reported the level of PM10 in Klong Luang district, Pathum Thani during December to April were 50–95 μg∙m^−3^, which were under levels of Thailand standard (120 μg∙m^−3^) but exceeded WHO guideline 2005 (50 μg∙m^−3^) [25].

Therefore, it is necessary to evaluate the health risks to children and adults from heavy metals in PM₂.₅ and PM₁₀ via inhalation exposure both the summer and wet sea-sons in the Pathum Thani Province, Thailand. Indeed, there are no published studies assessing the health effects from PM₂.₅ and PM₁₀ in this particular area. For example, Nuchdang et al. [13] studied the concentrations and the sources identification of PM₂.₅ and PM₁₀ using principal component and cluster analysis (PCA and CA) in the Pathum Thani Province, Thailand. An integrated study using a mixed approach can help more comprehensive understand the characteristics of heavy metals in PM₂.₅ and PM₁₀ concentration as well as possible health risks and concentration relationships [18]. Moreover, exceedance days in 2021 with the level of PM over the Thailand’s standard was also found in Pathum Thani province in the range of 1 – 30 days. Thailand’s Pollution Control Department reported that the average PM₂.₅ concentrations ranged from 10–40 µg∙m^−3^, with an overall national average of 22 µg∙m^−3^. For PM₁₀, the concentrations ranged from 19–99 µg∙m-3, with an average value of 40 µg∙m^−3^ [26]. In this study, PM2.5 and PM10 samples were collected at a Suburban Site in Pathum Thani, which was carried out secondary data on Nuchdang et al. [13]. The aims of this study as follow: (1) to assess the enrichment factors (EFs) to concern and identify heavy metals sources from PM_2.5_ and PM_10_ samples by distinguishing between geogenic and anthropogenic origins, (2) to assess the carcinogenic and non-carcinogenic risk posed to two age groups including, children and adults, via inhalation exposure. Our study helps to increasing awareness about air pollution control and risk mitigation strategies aimed at improving the ambient air quality in central area of Thailand.

## 2 Methods

### 2.1 Study area

The study was based on data from Nuchdang et al. [13], in which PM_2.5_ and PM_10_ samples were collected at a Suburban Site in Pathum Thani, Thailand. Pathum Thani Province is located in the central region of Thailand (14.24°N, 100.43°E), 6 m above mean sea level covering a total area of 1521.18 km^2^, accounting for 0.3 % of the country's total territory [25]. The total agricultural area is 842.22 km², accounting for 53.52 % of the total provincial area, especially rice farming covering of 65 % of the agricultural land (546.73 km^2^). In 2021, Pathum Thani Province had a total population of 1190,060. It ranked 4th in population density, with 779.92 people∙km^−2^, compared to Bangkok, which had the highest population density at 3523.9 people∙km^−2^[25], [27]. The GPP is 428,278 million THB, with 98.7 % contributed by the non-agricultural sector and only 1.3 % by the agricultural sector. Additionally, there are 3338 industrial factories in the province, comprising metallurgical and non-metallurgical industries (678 factories), followed by food (337 factories), plastics (301 factories), transportation (260 factories), chemicals (247 factories), wood products and furniture (191 factories), agriculture (71 factories), and others [25]. These figures highlight that Pathum Thani Province is one of the significant potential sources of particulate matter (PM₂.₅ and PM₁₀), indicating considerable emissions from agricultural activities (such as soil disturbance and post-harvest open burning in rice fields), transportation (road dust), and industrial operations (combustion processes) [13], [25]

For climate information, the average temperature was 29.92°C (Min–Max: 15.5–39.5°C), the relative humidity averaged 74.42 % (Min–Max: 47.5–94.3 %), and the total annual rainfall was 1382 mm [25]. The summary statistics, mean values of the meteorological parameters (Wind speed, relative Humidity, Air Pressure, Temperature, and rain fall) during the sampling period (18 February to 14 September 2021) are shown Table 1. The wet season showed higher mean values of the meteorological parameters than that summer season.

**Table 1:** Statistics values of meteorological parameters for the considered climatic season.

Variable	aWind Speed (m/s)	aRelative Humidity (mmHg)	aAir Pressure (mmHg)	aTemperature (°C)	aRain fall (mm)
Summer season					
Mean	0.4259	85.0159	755.8389	30.9147	3.8627
Minimum	0.0000	70.0000	753.5120	27.4000	0.0000
Maximum	2.5000	98.0000	758.5524	33.6000	74.8000
Standard Deviation	0.5708	8.2105	1.0458	1.3645	12.1033
Wet season					
Mean	0.7516	93.8431	757.5485	30.6873	8.6281
Minimum	0.2778	76.0000	754.2695	27.3000	0.1000
Maximum	1.3889	98.0000	763.0303	33.7500	71.6000
Standard Deviation	0.3268	4.6289	1.8626	1.4713	13.4871

a Data obtained from Nuchdang et al. [13]

### 2.2 Sample collection and methodology for elemental analysis

PM_2.5_ and PM_10_ samples were collected using a Tisch TE-Wilbur sampler and analyzed by Proton-Induced X-Ray Emission (PIXE), an analytical technique for determining elemental compositions [13]. Due to its ability to simultaneously detect nearly all elements with high sensitivity, PIXE analysis is widely applied in various fields, particularly in monitoring contamination in airborne dust [28]. The experimental and sampling site, where a Tisch TE-Wilbur sampler was used, was located approximately 2 m above ground level at the Thailand Institute of Nuclear Technology in Khlong Luang District, Pathum Thani Province, Thailand.

PM_2.5_ and PM_10_ sampling was conducted continuously from 18 February to 14 September 2021, covering the summer season (18 February–15 May) and the wet season (16 May–14 September). A total of 77 PM_2.5_ samples were collected—31 during the summer season and 46 during the wet season. For PM_10_, 49 samples were collected, with 21 from the summer season and 28 from the wet season [13]. The elemental analysis of PM₂.₅ and PM₁₀ included twelve elements: silicon (Si), sulfur (S), chlorine (Cl), potassium (K), calcium (Ca), titanium (Ti), chromium (Cr), manganese (Mn), iron (Fe), zinc (Zn), nickel (Ni), and copper (Cu).

### 2.3 Contamination assessment

The enrichment factor (EF) is a tool used to assess the extent of contamination in the environment. This tool is regarded as an effective method for identifying contaminant sources by distinguishing between geogenic and anthropogenic origins within the study area [16], [29].

(1) $$\text{EF }=\;\frac{{\left({\text{C}}_{\text{i}}/{\text{C}}_{\text{ref}}\right)}_{\text{aerosol}}}{{\left({\text{C}}_{\text{i}}/{\text{C}}_{\text{ref}}\right)}_{\text{crustal}}}$$

where Ci is the concentration of the studied element in the aerosol and crustal, respectively. Cref is the concentration of the reference element in the aerosol and crustal, respectively.

The Al, Fe, Sc and Ba are commonly used as reference in crustal materials due to its high natural abundance and relatively better chemical stability [30], [31]. In the present study, iron (Fe) was used as the reference metal. Due to the presence of many industries, including the productions of metal and non-metal industries, machinery manufacturing, and electrical appliance, approximately 1116 factories were established in Pathum Thani Province in 2021, representing 33.43 % of all industrial facilities in the province [25]. This finding is consistent with that of Nuchdang et al. (2023) [13], who reported relatively high concentrations of Fe and K in PM₂.₅ and PM₁₀. When the EF values approach unity (EF ≈ 1), the element is attributed mainly to natural rather than anthropogenic (human-related) sources. When the EF values exceed 10 (EF > 10), the element is considered to originate predominantly from non-crustal sources, such as industrial activities [16], [32].

### 2.4 Health risk assessment

The U.S. Environmental Protection Agency (EPA) method for health risk assessment was used in this study to evaluate the potential health effects from exposure to airborne metals and contaminants [33]. There are three types of exposure routes: inhalation, ingestion, and dermal contact. Human health risks assessments from inhalation are classified into two types: carcinogenic and non-carcinogenic of heavy metals. This study evaluated both carcinogenic and non-carcinogenic risks associated with heavy metals contaminating PM_2.5_ and PM_10_. The following subsections are the three steps in the process of evaluating health risk.

#### 2.4.1 Estimating toxicity values of exposure concentration both non-carcinogenic and carcinogenic risk

According to the US.EPA.’s Superfund program, the exposure concentration of metals via inhalation of atmospheric particles through mouth and nose was determined using the following equation for both non-carcinogen and carcinogen risk [16], [21].

For non-carcinogenic risk assessment, the concentration of metals was expressed as the average daily dose (ADD), representing typical short- or medium-term exposure, and was used to calculate the level of non-carcinogenic risk as follows.

(2) $$\text{ADD}\;=\;\frac{\text{C }\times \text{IR }\times \text{ ET}\times \text{ EF}\times \text{ED }}{\text{BW }\times {\text{AT}}_{\text{non cancer}}}$$

For carcinogenic risk assessment, the concentration of metals was converted into a lifetime average daily dose (LADD) or chronic daily intake (CDI), representing typical full life time exposure, which was then used to estimate the cancer risk using the following equation.

(3) $$\text{LADD }=\;\frac{\text{C }\times \text{IR }\times \text{ ET}\times \text{ EF}\times \text{ED }}{\text{BW }\times {\text{AT}}_{\text{cancer}}}$$

Where, ADD is the exposure concentration representing average daily dose (mg∙kg day^−1^), LADD is the exposure concentration representing lifetime daily dose (mg∙kg day^−1^), C is the concentration of the studied heavy metal in PM_2.5_ and PM_10_ (mg∙m^−3^), ET is the exposure time (24 hr∙day^−1^), EF is the exposure frequency (365 day∙year^−1^), ED is the exposure duration (year), 6 years for children and 24 years for adults, IR is the air inhalation rate (m^3^∙hr^−1^), 0.7 m^3^∙hr^−1^ for children and 0.9 m^3^∙hr^−1^ for adults, BW is body weight, 15 kg for children and 70 kg for adults, AT is the average time for carcinogen risk (AT_cancer_) and non-carcinogen risk (AT_non-cancer_), AT_non-cancer_children_ is ED (6 year)× 365 day∙year^−1^ × 24 hr∙day^−1^, AT_non-cancer_adults_ is ED (24 year)× 365 day∙year^−1^× 24 hr∙day^−1^, AT_cancer_children_ is 15 year× 365 day∙year^−1^, and AT_cancer_adults_ is 70 year× 365 day∙year^−1^.

#### 2.4.2 Estimating toxicity values for non-carcinogen effects

After calculating the ADD value, the non- cancer or carcinogenic target hazard quotient (THQ) is a tool to estimate the potential for non-carcinogenic health risk from exposure to a contaminant, developed by the US.EPA (1989, 2001) [34], [35]. The THQ value less than 1 (THQ < 1) indicates an acceptable or negligible non-carcinogenic health risk, whereas a value greater than 1 (THQ > 1) suggests a potential for adverse health effects and raises concerns regarding non-carcinogenic risks [9], [16], [21], [35].

(4) $${\text{THQ}}_{\text{i }}\;=\;\frac{\text{ADD }}{{\text{RfD}}_{\text{non cancer\_i}}}$$

Where, THQ_i_ is the non-carcinogenic risk associated with inhalation exposure to a single contaminant, assessed separately for each age group, i is the age group category such as children and adults, RfD_non cancer_i_ is the reference daily inhalation exposure for non-carcinogenic risk that is considered to have no adverse health effects over a lifetime (mg∙kg^−1^day^−1^) separately for each age group, RfD_non cancer_children_ is RfC × 7.6 m^3^∙day^−1^ × 1/15 kg, RfD_non cancer_adult_ is RfC × 20 m^3^∙day^−1^ × 1/70 kg, RfC is the inhalation reference concentration for chronic exposure (mg∙m⁻³), below which adverse non- carcinogenic health effects are unlikely to occur. The values are presented in Table 2.

**Table 2:** Values for the reference concentration for chronic inhalation exposure (RfC) and reference daily inhalation exposure for non-carcinogenic risk.

Substance Name	CASRN	RfC (mg∙m^−3^)	RfD_non cancer_children_ (mg∙kg^−1^∙day^−1^)	RfD_non cancer_adult_ (mg∙kg^−1^∙day^−1^)
bS	7783–06–4	c2 × 10^−3^	i5.7143 × 10^−4^	^i^1.0133 × 10^−3^
dCr	18540–29–9	e3 × 10^−5^	i.5200 × 10^−5^	^i^8.5714 × 10^−6^
Mn	7439–96–5	f5 × 10^−5^	i2.5333 × 10^−5^	^i^1.4286 × 10^−5^
gNi	7440–02–0	h5 × 10^−5^	i2.5333 × 10^−5^	^i^1.4286 × 10^−5^

b The assessment of sulfur (S) was considered based on the hydrogen sulfide (H₂S).

c Data obtained from U.S.EPA [38].

d The assessment of chromium (Cr) was considered based on the hexavalent chromium (Cr[V]).

e Data obtained from U.S.EPA [39].

f Data obtained from U.S.EPA [40].

g The assessment of nickel (Ni) was considered based on nickel refinery dust.

h Data obtained from U.S.EPA [41] and Vaio et al. [42].

i Data adapted from calculation.

To assess and compare the risk assessment results, we calculated the hazard index (HI), the total of THQ (TTHQ) of heavy metals, using Eq. (5).

(5) $${\text{HI}}_{\text{i}}\;=\;\sum_{\text{j}}^{\text{n}}{\text{THQ}}_{\text{i}}$$

Where, HI is the total hazard index, representing the sum of risks from the heavy metals considered, assessed separately for each age group, i is the age group category (e.g., children and adults), n is the total number of heavy metals, and j denotes a specific heavy metal. Similar to the THQ, HI value greater than 1 (HI > 1) indicates an increased likelihood of a toxicological response to the mixture of substances [36], [37].

#### 2.4.3 Estimating toxicity values for carcinogen effects

Cancer or carcinogenic risk (CR) represents the probability of cancer occurrence in an exposed population over a standard lifetime of 70 years. It is estimated by multiplying the chronic daily intake or LADD (from Eq. 2), by the cancer slope factors (CSF). The U.S. EPA considers carcinogenic risks in the range of 1× 10⁻⁶ to 1× 10⁻⁴ to be within the acceptable or tolerable range. A total carcinogenic risk exceeding 1 × 10⁻⁴ is considered unacceptable, whereas a lifetime carcinogenic risk below 1 × 10⁻⁶ is regarded as negligible [33], [34], [43]. The Integrated Risk Information System (IRIS) provides toxicity values and risk information for various chemicals, including cancer slope factors (see Table 2), which are used to estimate carcinogenic risk from exposure to specific substances, as shown below.

6) $${\text{CR}}_{\text{i}}\;=\text{ LADD }\times \;{\text{CSF}}_{\text{i}}$$

Where, CR is the probability of cancer, CSF is cancer slope factor (linear low-dose cancer potency factor) for the chemical (mg∙kg^−1^day^−1^), i is the age group category (e.g., children and adults), CSF_children_ is IUR× 15 kg/0.7 m^3^∙hr^−1^× 24 hr∙day^−1^, CSF_Adult_ is IUR× 70 kg/0.9 m^3^∙hr^−1^× 24 hr∙day^−1^, and IUR is reference values of inhalation unit risk from US.EPA database. The values were calculated using Eq. (7) and are presented in Table 3.

(7) $${\text{CSF}}_{\text{i}}\;=\text{ IUR }\times \frac{\text{BW }}{\;(\text{IR }\times \text{ET})}$$

**Table 3:** Values for inhalation unit risk (IUR) and the cancer slope factors for carcinogen risk.

Substance Name	CASRN	IUR (mg∙m^−3^)	CSF_children_ (mg∙kg^−1^∙day^−1^)	CSF_adult_ (mg∙kg^−1^∙day^−1^)
dCr	18540–29–9	e1.11 × 10^1^	^i^0.9911 × 10^1^	^i^3.5972 × 10^1^
gNi	7440–02–0	h2.40 × 10^−1^	i2.1429 × 10^−1^	i7.7778 × 10^−1^

d The assessment of chromium (Cr) was considered based on the hexavalent chromium (Cr[V]).

e Data obtained from U.S.EPA [39].

g The assessment of nickel (Ni) was considered based on nickel refinery dust.

h Data obtained from U.S.EPA [41] and Vaio et al. [42].

i Data adapted from calculation.

To assess and compare the risk assessment results, we calculated the sum of CR of heavy metals, using Eq. (8).

(8) $${\text{TCR}}_{\text{i}}\;=\;\sum_{\text{k}}^{\text{n}}{\text{CR}}_{\text{i}}$$

Where, TCR is the total carcinogenic risk, calculated as the sum of individual cancer risks from the heavy metals considered; n represents the total number of heavy metals, and k denotes a specific heavy metal. Similar to CR, a TCR value greater than 1 × 10⁻⁴ is considered unacceptable, whereas a lifetime cancer risk below 1 × 10⁻⁶ is regarded as negligible [33], [34], [43].

## 3 Results and discussion

### 3.1 PM_2.5_ and PM_10_ mass concentration

The summary data of PM₂.₅ and PM₁₀ mass concentrations were obtained at the Thailand Institute of Nuclear Technology, Khlong Luang District, Pathum Thani Province, Thailand. These data are available from Nuchdang et al. [13], and the statistical values for PM₂.₅ and PM₁₀ during the summer and wet seasons are presented in Table 4. The mean PM₁₀ concentration during the wet season exceeded the WHO standard 2021 [5] (45 µg∙m^−3^) and the EU standard 2005 [44] (50 µg∙m^−3^), but remained below the Thailand standard 2023 (80–120 µg∙m^−3^) and the U.S. EPA National Ambient Air Quality Standards (NAAQS) 2024 [45] (150 µg∙m^−3^). For PM₂.₅, the mean concentration during the summer season exceeded the WHO standard 2021 [5] (15 µg∙m^−3^) and the EU standard 2005 [44] (25 µg∙m^−3^), but remained below the U.S. EPA NAAQS [45] (35 µg∙m^−3^) and the Thailand standard 2023 [46] (50 µg∙m^−3^). In contrast, the mean PM₂.₅ concentration during the wet season exceeded all of the referenced standards.

**Table 4:** Statistics values for the sampling site during two (summer and wet season) climatic periods.

Variable	aPM_2.5_ (ug/m^3^) Summer season	aPM_2.5_ (ug/m^3^) Wet season	aPM_10_ (ug/m^3^) Summer season	aPM_10_ (ug/m^3^) Wet season
Minimum	1.6474	0.3885	12.7462	16.0554
Maximum	133.7261	174.2626	102.9984	242.0191
1st Quartile	9.7067	17.6966	27.9584	33.9855
Median	15.3043	84.3089	43.9693	82.0347
3rd Quartile	37.3025	119.3273	61.0987	107.9472
Mean	28.2205	77.5143	46.1518	86.0504

a Data obtained from Nuchdang et al. [13].

### 3.2 Metal concentrations

Data for twelve elements were retrieved from Nuchdang et al. [13], and the corresponding statistical values in PM₂.₅ and PM₁₀ during the summer and wet seasons are presented in Table 5, Table 6, respectively. The mean concentrations of seven elements, including S, K, Ca, Ti, Cr, Mn, and Fe, in PM₂.₅ and PM₁₀ during the summer season were significantly higher than those observed in the wet season.

**Table 5:** Statistics values for the 12 elements during two (summer and wet season) climatic periods of PM_2.5_.

	Si	S	Cl	K	Ca	Ti	Cr	Mn	Fe	Zn	Ni	Cu
Summer season: PM_2.5_ (ng/m^3^)												
Minimum	2.42	3.94	0.00	13.76	5.25	0.00	0.00	0.00	9.95	0.00	0.00	0.00
Maximum	60.26	107.92	30.25	355.01	100.36	125.04	948.16	125.81	3879.18	76.82	62.16	12.15
1st Quartile	6.73	14.71	2.35	41.66	12.81	0.00	0.00	0.00	22.10	0.00	0.00	0.00
Median	13.53	24.43	4.46	67.37	23.88	2.67	2.59	3.36	32.38	7.10	0.00	0.00
3rd Quartile	21.09	48.87	8.80	119.93	43.25	4.74	19.35	10.59	93.79	23.94	2.88	6.36
Mean	17.63	32.61	6.57	100.16	32.43	7.20	52.31	10.70	220.10	15.48	5.47	2.95
Wet season: PM_2.5_ (ng/m^3^)												
Minimum	1.08	0.71	0.00	1.23	1.51	0.00	0.00	0.00	0.00	0.00	0.00	0.00
Maximum	72.67	51.56	23.82	276.52	137.64	39.81	444.20	82.17	1381.16	75.00	94.06	35.08
1st Quartile	4.28	2.74	1.31	16.88	9.85	0.00	0.00	0.00	16.95	0.00	0.00	0.00
Median	10.55	10.83	3.17	46.72	16.66	1.44	1.73	0.00	27.92	7.76	0.00	0.00
3rd Quartile	30.70	17.45	7.90	72.65	44.21	3.60	6.30	6.42	78.11	16.52	5.80	5.75
Mean	18.09	12.77	5.78	57.31	29.52	3.02	27.94	6.26	107.76	13.34	7.51	3.49

**Table 6:** Statistics values for the 12 elements during two (summer and wet season) climatic periods of PM_10_.

	Si	S	Cl	K	Ca	Ti	Cr	Mn	Fe	Zn	Ni	Cu
Summer season: PM_10_ (ng/m^3^)												
Minimum	2.05	4.40	3.17	28.68	33.10	1.42	0.00	0.00	38.69	0.00	0.00	0.00
Maximum	42.09	47.48	35.89	220.67	261.08	30.12	1033.32	124.40	4145.35	58.34	595.56	18.60
1st Quartile	7.83	13.37	5.22	51.00	122.49	6.52	0.00	0.00	71.95	9.24	0.00	0.00
Median	11.02	23.99	10.23	77.81	156.71	11.23	3.27	3.27	138.22	17.55	0.00	0.00
3rd Quartile	16.92	41.07	20.88	118.76	176.52	18.25	13.85	7.25	259.18	35.90	5.38	7.76
Mean	14.00	26.33	12.90	92.50	158.09	12.11	68.48	11.55	378.47	21.26	35.34	3.77
Wet season: PM_10_ (ng/m^3^)												
Minimum	1.67	1.44	1.20	10.07	29.18	0.00	0.00	0.00	22.42	0.00	0.00	0.00
Maximum	57.71	50.05	86.20	185.43	485.94	37.60	490.07	62.25	1753.12	82.08	233.94	53.34
1st Quartile	5.36	4.07	4.11	23.14	54.20	1.52	0.00	1.84	39.77	8.16	0.00	0.00
Median	9.73	7.84	8.29	39.52	77.07	3.92	0.00	4.48	86.17	17.35	0.00	0.00
3rd Quartile	24.22	15.94	18.89	74.27	165.36	12.72	4.82	7.46	162.37	27.05	4.44	6.54
Mean	16.03	11.54	17.26	55.27	125.58	7.49	44.39	9.91	229.92	22.78	15.78	4.99

The concentrations of ten elements (Si, S, Cl, K, Ca, Ti, Cr, Mn, Fe, and Zn) in PM₂.₅ during the summer season exceeded the European Union regulatory limit (5 ng∙m^−3^) as referenced by World Health Organization [47], by ranging from 1.31 to 44.02 times. In the wet season, the exceedance ranged from 1.25 to 21.55 times, except for Ti, which remained below the limit. Similarly, in PM₁₀, exceedances during the summer season ranged from 2.31 to 75.69 times, including Ni, which exceeded its limit (20 ng∙m^−3^) [47] by 1.77 times. In the wet season, exceedances including Cu, ranged from 3.72 to 829.07 times.

### 3.3 Levels of heavy metal in PM_2.5_ and PM_10_

**Fig. 1:**
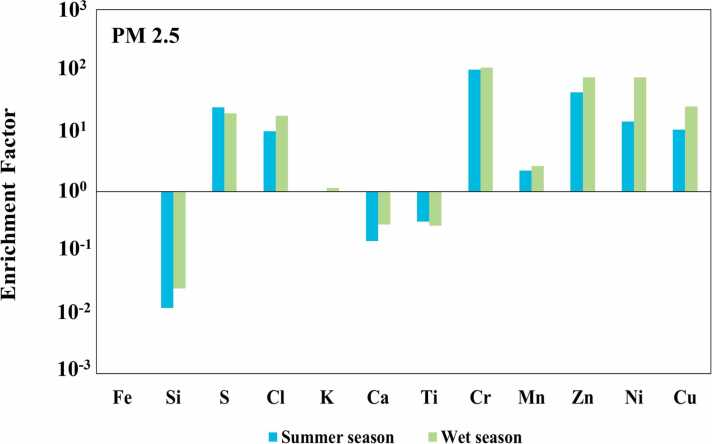
The crustal EF for the element based on average seasonal metal concentration during two (summer and wet season) climatic periods of PM_2.5_.

**Fig. 2:**
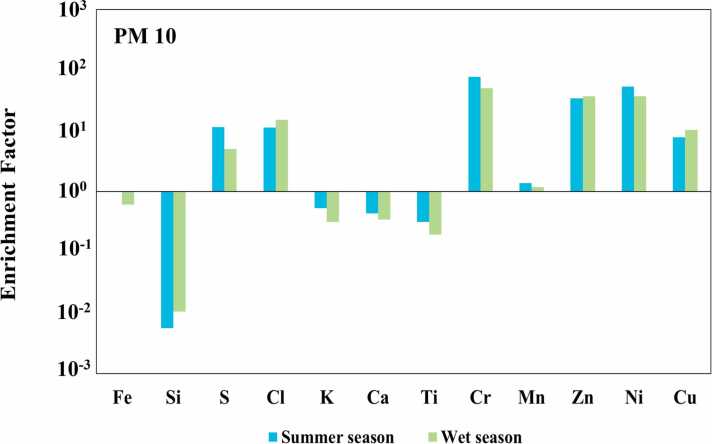
The crustal EF for the element based on average seasonal metal concentration during two (summer and wet season) climatic periods of PM_10._

The EF values for each toxic metal in PM₂.₅ and PM₁₀ were analyzed for two climatic periods, including summer and wet seasons. The seasonal variations in EF provide insight into the potential sources of metals, whether natural or anthropogenic. The detailed EF values for each metal and season are presented in Fig. 1, Fig. 2.

For Si, K, Ca, Ti, Mn, and Fe, low EFs ranging from 0.05 to 3 were also observed in PM₁₀ and PM₂.₅ particles, indicating that the concentrations of these elements are primarily influenced by natural crustal sources, such as soil and mineral dust [48]. These findings are consistent with the results reported by Tasic et al. [49], Diana et al. [50] and Ahmad et al. [12], who observed the EFs for various elements in PM₂.₅ and PM₁₀ ranging from 0.01 to 10, further supporting their classification as crustal in origin. The EF values between 10 and 100 were observed for S, Cl, Zn, Ni, and Cu in PM₂.₅ and PM₁₀ both climatic periods, except for S during the wet season. These values indicate significant contributions from non-crustal sources, such as fuel combustion, traffic emissions, and industrial processes. Similar findings were reported by Mohammed et al. [48], Rai et al. [51], and Gao et al. [52], where EF values of Zn, Ni, and Cu ranged from 10 to 100 in PM₂.₅, reflecting the accumulation of heavy metals in fine particulate matter from non-crustal sources or anthropogenic activities. Furthermore, Nayebare et al. [53] reported EF values greater than 10 for Ni, Cl, and S, further supporting the influence of anthropogenic sources such as fuel combustion and industrial operations. Cr exhibited the highest enrichment factor values (>100) in PM₂.₅ across all seasons, while showing high values ranging from 51 to 78 in PM₁₀. These findings are agree well with the results reported by Gao et al. [52] and Varrica and Alaim [54], who observed higher EF values of Cr in PM₂.₅ than in PM₁₀, indicating contributions from industrial activities and traffic emissions. The previous study by Nuchdang et al. [13] have sugested that Mn, Fe, Cr and Ni represented a mixture of road dust and industrial emission sources, particularly Mn, Fe and Cr are elements for road dust sources Onat et al. [55] and Bie et al. [56].

### 3.4 Health risk assessment

#### 3.4.1 Non-carcinogenic risk analysis

The non-carcinogenic and carcinogenic risks associated with toxic metals in PM₂.₅ and PM₁₀ were analyzed. For non-carcinogenic risk, the THQ and values of S, Cr, and Mn were investigated and reported in Table 7, Table 8. Based on the average across climatic periods (summer and wet seasons), age groups (children and adults), and both PM₂.₅ and PM₁₀, the order of average risk values from highest to lowest was Cr > Mn > S. The average non-carcinogenic risk values associated with toxic metals in PM₂.₅ ranged from 0.00028 to 0.07525 for adults and from 0.00197 to 0.53903 for children. For PM₁₀, the values ranged from 0.00025 to 0.09850 for adults and from 0.00178 to 0.70560 for children. The calculated HI values ranged from 0.0459 to 0.1090 for adults and from 0.3285 f to 0.7811 for children. All THQ values for individual metals were below 1, and the cumulative non-carcinogenic risk (HI) also remained under the threshold of 1, indicating an acceptable risk level. According to the U.S. EPA [34], an THQ value below 1 is considered to represent a non-significant risk. Because several parameters (see Eq. 2) related to children's behavior and physiology, the ratio of air inhalation rate to body weight (IR/BW) for children is 0.047 m³ ·hr⁻¹ ·kg⁻¹ , which is higher than that for adults (0.013 m³·hr⁻¹·kg⁻¹), resulting in greater toxicant intake per kilogram of body weight in children. The individual and cumulative non-carcinogenic risk values for children is significantly higher than adults, which increase level of toxic exposure. Therefore, the results of this study were consistent with other studies conducted in Italy [20], [50], Nigeria [57], Mexico [21], and Ethiopia [43].

**Table 7:** Non-carcinogenic target hazard quotient (THQ) for sulfur (S), chromium (Cr), and manganese (Mn) during two (summer and wet season) climatic periods for each receptor (adult and children).

Chemicals	Age group	THQ			
		Min.	Median	Mean	Max.
Summer season: PM_2.5_					
S	Adults	0.000085	0.000527	0.000704	0.002328
	Children	0.000609	0.003775	0.005041	0.016680
Cr	Adults	0.00	0.003729	0.075245	1.363792
	Children	0.00	0.026713	0.539034	9.769853
Mn	Adults	0.00	0.002904	0.009233	0.108579
	Children	0.00	0.020802	0.066142	0.777830
Wet season: PM_2.5_					
S	Adults	0.000015	0.000234	0.000275	0.001113
	Children	0.000110	0.001673	0.001973	0.007970
Cr	Adults	0.00	0.002495	0.040181	0.638921
	Children	0.00	0.017874	0.287848	4.577066
Mn	Adults	0.00	0.00	0.005406	0.070910
	Children	0.00	0.00	0.038725	0.507982
Summer season: PM_10_					
S	Adults	0.000095	0.000518	0.000568	0.001024
	Children	0.000679	0.003708	0.004069	0.007339
Cr	Adults	0.00	0.004702	0.098496	1.486286
	Children	0.00	0.033685	0.705601	10.647372
Mn	Adults	0.00	0.002821	0.009967	0.107357
	Children	0.00	0.020207	0.071398	0.769078
Wet season: PM_10_					
S	Adults	0.000031	0.000169	0.000249	0.001080
	Children	0.000222	0.001212	0.001783	0.007735
Cr	Adults	0.00	0.00	0.063855	0.704888
	Children	0.00	0.00	0.457438	5.049636
Mn	Adults	0.00	0.003867	0.008549	0.053724
	Children	0.00	0.027706	0.061243	0.384868

**Table 8:** Non-carcinogen target hazard quotient (THQ) and total target hazard quotient (TTHQ) or hazard index (HI) values for sulfur (S), chromium (Cr), and manganese (Mn) during two (summer and wet season) climatic periods for each receptor (adults and children*)*.

Climatic periods	Age group	jTHQ			kHI
		S	Cr	Mn	
Summer season: PM_2.5_	Adults	0.000704	0.075245	0.009233	0.085182
	Children	0.005041	0.539034	0.066142	0.610217
Wet season : PM_2.5_	Adults	0.000275	0.040181	0.005406	0.045862
	Children	0.001973	0.287848	0.038725	0.328546
Summer season: PM_10_	Adults	0.000568	0.098496	0.009967	0.109031
	Children	0.004069	0.705601	0.071398	0.781068
Wet season : PM_10_	Adults	0.000249	0.063855	0.008549	0.072653
	Children	0.001783	0.457438	0.061243	0.520464

^j^THQ is target hazard quotient

k HI, hazard index (TTHQ, total target hazard quotient)

j THQand ^k^HI < 1, no health risk; ^j^THQ and ^k^HI ≥ 1 indicates potential health risk [34].

#### 3.4.2 Lifetime average daily dose (LADD) and carcinogenic risk (CR) analysis

LADD was evaluated separately for children and adults based on inhalation intake of Cr and Ni during the summer and wet seasons, as reported in Table 9. The average LADD values for Cr in PM₂.₅ for adults during the summer and wet seasons were 2.2113 × 10⁻⁷ and 1.1808 × 10⁻⁷, respectively, while for children the values were 9.3638 × 10⁻⁷ and 5.0003 × 10⁻⁷. For PM₁₀, the LADD values for Cr during the summer and wet seasons were 2.8946 × 10⁻⁷ and 1.8765 × 10⁻⁷ for adults, and 1.2257 × 10⁻⁶ and 7.9464 × 10⁻⁷ for children, respectively. For Ni, the average LADD values in PM₂.₅ for adults during the summer and wet seasons were 6.5422 × 10⁻⁸ and 3.1749 × 10⁻⁸, while for children the values were 2.7703 × 10⁻⁷ and 1.3444 × 10⁻⁷, respectively. For PM₁₀, the LADD values for Ni during the summer and wet seasons were 1.4937 × 10⁻⁷ and 6.6683 × 10⁻⁸ for adults, and 6.3253 × 10⁻⁷ and 2.8237 × 10⁻⁷ for children, respectively. The results of this study show that LADD values were higher during the summer season compared to the wet season, which is consistent with findings by Breton et al. [21] in Mexico and Demissie et al. [43] in Ethiopia. Additionally, children exhibited higher LADD values than adults, aligning with findings from Ethiopia [43], Pakistan [58], and Bangladesh [59]. For carcinogenic risk, the values of Cr and Ni were investigated and reported in Table 7, Table 8. For both PM₂.₅ and PM₁₀, the order of carcinogenic risk values for both adults and children was Cr > Ni, based on the average across climatic periods (summer and wet seasons) and age groups (children and adults). According to the U.S. EPA [60], the individual and total carcinogenic risk values of Cr and Ni were within the acceptable range of 10⁻⁶ to 10⁻⁴, indicating that the associated cancer risk, between 1 in 1,000,000 and 1 in 10,000, is considered negligible. The average carcinogenic risk values from toxic metals in PM₂.₅ ranged from 5.0884 × 10⁻⁸ to 7.9544 × 10⁻⁶ for adults and from 5.9364 × 10⁻⁸ to 9.2802 × 10⁻⁶ for children. In the case of PM₁₀, the values ranged from 5.1865 × 10⁻⁸ to 1.0412 × 10⁻⁵ for adults and from 6.0509 × 10⁻⁸ to 1.2148 × 10⁻⁵ for children. Similar to the non-carcinogenic risk, the average carcinogenic risk values for children were significantly higher than those for adults. This finding is consistent with studies by Demissie et al. [43] in Ethiopia, Bello et al. [57] in Nigeria, and Wahil et al. [61] in Malaysia. Overall, the carcinogenic risk from toxic metals in PM₂.₅ and PM₁₀ observed in this study was within the acceptable range. These findings are consistent with those reported in other studies conducted in China [16], Italy [20], Nigeria [57], Mexico [21], and Thailand at Ari in Bangkok [9]. Carcinogenic risk associated with PM₂.₅ and PM₁₀ is lung cancer [62], [63], [64], chronic obstructive pulmonary disease (COPD), idiopathic pulmonary fibrosis (Johannson et al., 2014) [65], risk for symptoms of acute respiratory infections (ARIs) [66], pneumonia and pneumonia mortality rate [67], asthma [68] and heart disease [69], [70]. Fore long term exposure to PM₂.₅ and PM₁₀, children have higher risks than adults in the respiratory infections, abnormal lung development, asthma, and infant mortality due to their developing organs, higher breathing rates, and immature immune systems [68], [69], [71]. Whereas adults are more susceptible to chronic diseases in COPD, heart disease, and lung cancer [69], [72]. PM₂.₅ is considered higher prevalent and hazardous than PM₁₀ in terms of inflammatory and carcinogenic properties, contributing more harmful to human health for the long term due to its smaller size and deeper penetration into the respiratory system, including the alveoli and bloodstream [4], [69], [73]. Moreover, it is more persistent in the environment [4], [69]. Overall, the non-carcinogenic risks of S, Cr, and Mn, as well as the carcinogenic risks of Cr and Ni, remained within acceptable levels in Pathum Thani. Nonetheless, enhanced attention from researchers, environmental practitioners, and policymakers is essential to effectively control toxic metal pollution in PM₂.₅ and PM₁₀ and to support evidence-based air quality management.

**Table 9:** Carcinogenic risk (CR) level and lifetime average daily dose (LADD) for chromium (Cr) and nickel (Ni) during two (summer and wet season) climatic periods of PM_2.5_ and PM_10_ for each receptor (adult and children*)*.

Chemicals	Age group	CR				LADD			
		Min.	Median	Mean	Max.	Min.	Median	Mean	Max.
Summer season: PM_2.5_									
Cr	Adults	0.00	3.9421 × 10^−7^	7.9544 × 10^−6^	1.4417 × 10^−4^	0.00	1.0959 × 10^−8^	2.2113 × 10^−7^	4.0079 × 10^−6^
	Children	0.00	4.5991 × 10^−7^	9.2802 × 10^−6^	1.6820 × 10^−4^	0.00	4.6405 × 10^−8^	9.3638 × 10^−7^	1.6972 × 10^−5^
Ni	Adults	0.00	0.00	5.0884 × 10^−8^	2.5257 × 10^−7^	0.00	0.00	6.5422 × 10^−8^	3.2473 × 10^−7^
	Children	0.00	0.00	5.9364 × 10^−8^	2.9466 × 10^−7^	0.00	0.00	2.7703 × 10^−7^	1.3751 × 10^−6^
Wet season: PM_2.5_									
Cr	Adults	0.00	2.6377 × 10^−7^	4.2477 × 10^−6^	6.7543 × 10^−5^	0.00	7.3325E−09	1.1808 × 10^−7^	1.8776 × 10^−6^
	Children	0.00	3.0773 × 10^−7^	4.9557 × 10^−6^	7.8800 × 10^−5^	0.00	3.1050E−08	5.0003 × 10^−7^	7.9510 × 10^−6^
Ni	Adults	0.00	0.00	1.1421 × 10^−6^	1.4302 × 10^−5^	0.00	0.00	3.1749 × 10^−8^	3.9760 × 10^−7^
	Children	0.00	0.00	1.3324 × 10^−6^	1.6686 × 10^−5^	0.00	0.00	1.3444 × 10^−7^	1.6836 × 10^−6^
Summer season: PM_10_									
Cr	Adults	0.00	4.9709 × 10^−7^	1.0412 × 10^−5^	1.5712 × 10^−4^	0.00	1.3819 × 10^−8^	2.8946 × 10^−7^	4.3679 × 10^−6^
	Children	0.00	5.7993 × 10^−7^	1.2148 × 10^−5^	1.8331 × 10^−4^	0.00	5.8516 × 10^−8^	1.2257 × 10^−6^	1.8496 × 10^−5^
Ni	Adults	0.00	0.00	1.1618 × 10^−7^	1.9580 × 10^−6^	0.00	0.00	1.4937 × 10^−7^	2.5174 × 10^−6^
	Children	0.00	0.00	1.3554 × 10^−7^	2.2843 × 10^−6^	0.00	0.00	6.3253 × 10^−7^	1.0660 × 10^−5^
Wet season: PM_10_									
Cr	Adults	0.00	0.00	6.75035 × 10^−6^	7.4516 × 10^−5^	0.00	0.00	1.8765 × 10^−7^	2.0715 × 10^−6^
	Children	0.00	0.00	7.87541 × 10^−6^	8.6936 × 10^−5^	0.00	0.00	7.9464 × 10^−7^	8.7719 × 10^−6^
Ni	Adults	0.00	0.00	5.1865 × 10^−8^	7.6913 × 10^−7^	0.00	0.00	6.6683 × 10^−8^	9.8889 × 10^−7^
	Children	0.00	0.00	6.0509 × 10^−8^	8.9732 × 10^−7^	0.00	0.00	2.8237 × 10^−7^	4.1875 × 10^−6^

**Table 10:** Carcinogenic risk (CR) and total carcinogenic risk (TCR) values for chromium (Cr) and manganese (Mn) during two (summer and wet season) climatic periods for each receptor (adult and children*)*.

Climatic periods	Age group	LCR		MTCR
		Cr	Ni	
Summer season: PM 2.5	Adults	7.9544 × 10^−6^	5.0884 × 10^−8^	8.0053 × 10^−6^
	Children	9.2802 × 10^−6^	5.9364 × 10^−8^	9.3396 × 10^−6^
Wet season : PM 2.5	Adults	4.2477 × 10^−6^	1.1421 × 10^−6^	5.3898 × 10^−6^
	Children	4.9557 × 10^−6^	1.3324 × 10^−6^	6.2881 × 10^−6^
Summer season: PM 10	Adults	1.0412 × 10^−5^	1.1618 × 10^−7^	1.0528 × 10^−5^
	Children	1.2148 × 10^−5^	1.3554 × 10^−7^	1.2284 × 10^−5^
Wet season : PM 10	Adults	6.75035 × 10^−6^	5.1865 × 10^−8^	6.8022 × 10^−6^
	Children	7.87541 × 10^−6^	6.0509 × 10^−8^	7.9359 × 10^−5^

^L^CR and ^M^TCR < 1 × 10^−6^*,* indicate low potential carcinigenic risk to human health [33].

^L^CR and ^M^TCR > 1 × 10^−4^, indicates potential carcinogenic risk to human health [33].

1 × 10^−6^ < ^L^CR and ^M^TCR < 1 × 10^−4^, indicate acceptable level of carcinogenic risk to human health [33].

L CR is carcinogen risk

M TCR is total carcinogen risk

## 4 Conclusions

This study determined the statistical values of PM₂.₅ and PM₁₀ mass concentrations and the concentrations of twelve elements including, Si, S, Cl, K, Ca, Ti, Cr, Mn, Fe, Zn, Ni, and Cu, in PM₂.₅ and PM₁₀ during the summer and wet seasons. The analysis was based on data from Nuchdang et al. [13], conducted at the Thailand Institute of Nuclear Technology, Khlong Luang District, Pathum Thani Province. Subsequently, enrichment factor (EF) with both carcinogenic and non-carcinogenic health risks associated with heavy metals in PM₂.₅ and PM₁₀ were evaluated.

The findings revealed that the EF values of Zn, Ni, and Cu ranged from 10 to 100, while Cr exhibited the highest EF values, ranging from 51 to 111. These values indicate significant contributions from non-crustal sources, such as fuel combustion, traffic emissions, and industrial processes. The results of this study highlight the PM₂.₅ and PM₁₀ mass concentrations during the wet season as causes for concern regarding public health in the study area. The findings indicated that the mean concentration of PM₂.₅ exceeded the standards set by the WHO, EU, U.S. EPA NAAQS, and Thailand. In contrast, PM₁₀ concentrations exceeded the WHO and EU standards but remained below the thresholds established by the Thailand standard and U.S. EPA NAAQS. The hazard quotient (HQ) and the hazard index (HI) for individual metals, including S, Cr, and Mn, via inhalation exposure among children and adults during both the summer and wet seasons were below 1. This indicates an acceptable risk level and suggests no potential non-carcinogenic health risk for residents at the Suburban Site in Pathum Thani. Similarly, the incremental lifetime carcinogenic risk from inhalation exposure among children and adults in both seasons ranged from 10⁻⁶ to 10⁻⁴, which is considered acceptable according to international health risk assessment guidelines. Although overall health risks were within acceptable limits, the analysis consistently showed higher risk values for children compared to adults, especially for respiratory infections, abnormal lung development, asthma, and infant mortality. This finding suggests that children are more vulnerable to health risks associated with toxic metals in PM₂.₅ and PM₁₀ via inhalation exposure in the study area. Therefore, it is essential to implement targeted and effective pollution control policies to mitigate toxic metal pollution in PM₂.₅ and PM₁₀ and to intensify air quality management strategies, with particular emphasis on safeguarding children's health.

## Additional information

No additional information is available for this paper.

## CRediT authorship contribution statement

**Unchalee Suwanmanee:** Writing – review & editing, Writing – original draft, Validation, Supervision, Project administration, Methodology, Investigation, Formal analysis, Conceptualization. **Dussadee Rattanaphra:** Writing – original draft, Visualization, Validation, Resources, Methodology, Data curation, Conceptualization. **Sasikarn Nuchdang:** Resources, Data curation. **Kittiwan Kitpakornsanti:** Resources, Data curation. **Sittinun Tawkaew:** Resources, Data curation. **Wilasinee Kingkam:** Resources, Data curation.

## Funding sources

We gratefully acknowledge financial support from the Thailand Institute of Nuclear Technology (2024–2025 University Fund) and the Faculty of Engineering, Srinakharinwirot University.

## Declaration of Competing Interest

The authors declare that they have no known competing financial interests or personal relationships that could have appeared to influence the work reported in this paper.

## Data Availability

Data will be made available on request.
